# Functional Val66Met polymorphism of Brain-derived neurotrophic factor in type 2 diabetes with depression in Han Chinese subjects

**DOI:** 10.1186/1744-9081-9-34

**Published:** 2013-08-22

**Authors:** Jian-Xin Zhou, He-Chao Li, Xue-Jun Bai, Bao-Cheng Chang, Chun-Jun Li, Pei Sun, Li-Ming Chen

**Affiliations:** 1 2011 Collaborative Innovation Center of Tianjin for Medical Epigenetics, Key Laboratory of Hormone and Development (Ministry of Health), Metabolic Disease Hospital & Tianjin Institute of Endocrinology, Tianjin Medical University, Tianjin, China; 2 Center of Psychology and Behavior, Tianjin Normal University, Tianjin, China

**Keywords:** Type 2 diabetes (T2DM), Depression, Brain-derived neurotrophic factor (BDNF), Polymorphism

## Abstract

**Background:**

Brain-derived neurotrophic factor (BDNF) has been implicated in the pathogenesis of major depression. Individuals with type 2 diabetes (T2DM) have a high prevalence of major depression and low levels of BDNF. We therefore explored whether the BDNF Val66Met polymorphism is associated with co-morbid depression and whether depression affects the serum levels of BDNF in a Han Chinese subjects with T2DM.

**Methods:**

A Total of 296 T2DM patients and 70 healthy volunteers (Health control, HC group) were recruited in this study. T2DM patients were divided into two subgroups: depressive diabetes group (DDM group, n = 64) and non-depressive diabetes group (NDDM group, n = 232), according to the presence or the absence of depression assessed by Center for Epidemiologic Studies Depression Scale (CES-D) and Patient Health Questionnaire-9 (PHQ-9). Val66Met polymorphism was detected by polymerase chain reaction-restriction fragment length polymorphism analysis (PCR-RFLP). Serum BDNF levels were measured by ELISA kit.

**Results:**

In this study, 21.6% (64/296) patients with T2DM had depression. The BDNF Val66Met genotype distributions were statistically different among the three groups (χ^2^ = 7.39, p < 0.05). DDM group carried the highest frequencies of Met allele (53.9%) compared to HC group (39.3%) and NDDM group (38.8%). Subjects with Met/Met had lowest serum BDNF levels (76.59 ± 5.12 pg/ml, F = 7.39, p < 0.05) compared to subjects with Val/Met (79.04 ± 5.19 pg/ml) and Val/Val (83.83 ± 3.97 pg/ml). Within T2DM group, it was also observed that the serum BDNF levels in DDM group were significantly lower than those in NDDM group (76.67 ± 5.35 vs. 79.84 ± 3.97 pg/ml, p < 0.05). In type 2 diabetes subjects, BDNF serum levels were significant correlations with genotypes (r = −0.346, p < 0.01), depression scores (r = −0.486, p < 0.01) and HbA1c (r = −0.168, p < 0.05). After adjustment for gender, HbA1c, BMI and numbers of complications, BDNF Val/Met genotype distributions (OR = 2.105, p < 0.05) and decreased serum BDNF levels (OR = 0.835, p < 0.01) were independently associated with depression in T2DM.

**Conclusions:**

The BDNF Val66Met polymorphism might be implicated in the pathogenesis of depression in T2DM by decreasing serum BDNF levels in Han Chinese Subjects.

## Introduction

Depression and type 2 diabetes (T2DM) are two of the most prevalent and devastating diseases. There is a strong association between T2DM and depression [1,2]. Epidemiological data indicates that approximately 26-30% of diabetic patients suffer from differential severity of depression, excessive above that of the normal population [3,4]. Moreover, the mortality rate is significantly higher among patients with T2DM and depression than among patients with diabetes without depression [5]. Therefore, it is important to study the etiology of depression with T2DM.

Brain-derived neurotrophic factor (BDNF) is a member of the neurothophic factor family, which plays a key role in regulating survival, growth and maintenance of neurons [6]. It has been suggested that reduction in BDNF expression is a pathogenic factor common to Alzheimer’s disease and major depression [7,8]. For example, a study by Karege et al. have reported that major depressive patients exhibited significantly lower levels of serum BDNF compared with normal controls [9,10], whereas the use of antidepressants led to an up regulation of BDNF in the hippocampus of subjects with major depression [8]. The association between BDNF and depression is also supported by animal studies in which infusion of recombinant BDNF exerted antidepressant effect [11].

The human BDNF gene has been mapped to chromosome 11p13 and a common single nucleotide polymorphism (SNP) consisting of a missense change (G196A), which produces a valine (Val) to methionine (Met) change, has been identified in the coding exon of the BDNF gene at position 66 (Val66Met) [12]. The Val66Met polymorphism in the BDNF gene has been shown to impact intracellular trafficking and activity-dependent secretion of BDNF [13]. Clinical studies demonstrate that the Met allele is associated with decreased hippocampal volume in both normal and depressed patients and with decreased executive function and cognition [13,14] Hong et al. first reported that BDNF Val66Met polymorphism was associated with major depressive disorders in a Caucasian population [15]. Subsequently, increasing clinical studies have confirmed that the Met allele was more commonly found among individuals with mood disorders [16-18]. In line with these clinical findings, animal study showed that a variant BDNF mouse (BDNF Met/Met) reproduced the phenotypic hallmarks of humans with this variant allele, and exhibited increased anxiety-related behaviors [19]. Paradoxically, Tsai SJ et al. reported a higher incidence of depression in Val, not Met carriers [20]. Currently, it is still unclear what the consequences of the Val66Met polymorphism are on the brain function, as both alleles have been associated with different disease processes, including emotion response [21], esoghageal hypersensitivity [22], individual's sexual activities [23] and depression [15,24,25]. Of interest, several studies have shown that the degree of therapeutic response to antidepressants is associated with the BDNF Val66Met polymorphism, suggesting that BDNF gene may be a good candidate gene for the pharmacogenetic study of antidepressants [7].

Furthermore, a decrease in BDNF concentration has been strongly linked to T2DM. BDNF was reported to improve glucose metabolism insulin sensitivity, and reduce food consumption [26]. The direct role of BDNF in metabolism is supported by studies on BDNF-deficient mice, which developed diabetes and obesity in early adulthood [27]. Animals studies have also shown that BDNF has important effects on the regulation of eating behavior [28] and modulation of the secretion and activities of insulin, leptin, ghrelin and pro-inflammatory cytokines associated with energy homeostasis[29]. These findings suggest that BDNF may also play a pathogenic role in the development of obesity and T2DM in humans. More recent epidemiologic studies [30] showing T2DM patients develop more depression and at the same time have lower serum BDNF levels compared to non-diabetic individuals [28,31,32], posed an interesting question whether the higher rate of depression in diabetes may be in part mediated by decreased BDNF levels. However, the clinical significance of the effect of BDNF levels on depression and the impact of Val66Met polymorphism remain unclear in T2DM patients with depression. Thus, BDNF genetic studies of patients with and without depression may shed light on the potential genetic link between depression and T2DM. We therefore hypothesized that a genetic variation (Val to Met substitution) in BDNF leading to a reduction in serum BDNF levels in T2DM may contribute to depression and diabetes. In this study, we investigated whether the Val66Met polymorphism and serum BDNF levels are different in Han Chinese subjects who suffered from T2DM with or without depression.

## Subjects and Methods

### Participants

All subjects were of Chinese origin (Han Chinese) and lived in the same region at the time of the study. A total of 296 T2DM patients from Metabolic Disease Hospital of Tianjin Medical University were recruited into this study. T2DM was diagnosed according to the 1999 WHO criteria. In the ethnicity, age and gender-matched control group, 70 healthy people without mental illness, family history of psychiatric disorders and T2DM were recruited from the same area. All T2DM subjects were divided into two subgroups: depressive group (DDM group, n = 64) and non-depressive group (NDDM group, n = 232), depending on presence or absence of depression. This study was approved by the local ethics committee review board and was conducted using Good Clinical Practice in accordance with the Declaration of Helsinki. All subjects gave written informed consent before entering the study.

### Depression assessment

The Center for Epidemiologic Studies Depression Scale (CES-D) Chinese edition [33] was used to assess depressive symptoms. This scale is the most widely used depression screening scale. The Chinese version of the CES-D scale shows good reliability and validity across all ages in urban and rural population [34]. This scale consists of 16 negative affect and 4 positive affect items, such as "I felt depressed", "I felt lonely", and "I was happy". Participants were asked about the number of days on which they experienced depressive symptoms during the previous week. Each item was accompanied by a standard four-point Likert scale of potential responses: 1 = none, 2 = one or two days a week, 3 = three or four days per week, and 4 = five days or more per week. Higher scores on the CES-D indicate more depressive symptoms. In the scale, four items that describe positive effects were reversed before conducting our analysis. Radloff has suggested that a score of 16 should be the cut-off point for the scale [35], while other studies have adopted 21 or 22 as the cut-off point [36]. Given the complexity of the cutoff point selection, this study used CES-D scale combined with another scale named Patient Health Questionnaire-9 (PHQ-9) [37] to assess to be depressed together. Participants rated the frequency of experiencing nine symptoms during the previous two weeks: 0) not at all, 1) on several days, 2) on more than half of the days, and 3) nearly every day. The PHQ-9 scores range from 0 to 27, with higher scores indicating high severity of symptoms. The CES-D scale score cut-off point was set to 16, and the PHQ-9 scale was set to 5. The recruited subjects were asked to complete the 2 questionnaires by themselves at different days. Subjects meeting the criteria of both scales were defined as depression.

### BDNF polymorphism analyses

Genomic DNA was extracted from peripheral whole blood of subjects by proteinase K digestion. BDNF-Val66Met polymorphism was performed by polymerase chain reaction-restriction fragment length polymorphism (PCR-RFLP). DNA fragments of interest were amplified by polymerase chain reaction (PCR) with the primers 5'-ACTCTGGAGAGCGTGAAT-3' and 5'-ATACTGTCACACACGCTC-3' designed by Oligo 6.0 primer analysis software. A total volume of 25 μl PCR reaction contains 2 μl of genomic DNA, 2.5 μl of 10 × PCR buffer, 2 μl of dNTP, 1 μl of each primer, 16.2 μl of distilled water and 0.3 μl of Taq polymerase. The PCR conditions were as follows: predegeneration at 94°C for 5 min, 35 cycles of 30s at 94°C, annealing at 55°C for 30s and extending at 72°C for 1 min. The thermal cycle was completed with 72°C for 10 min. The Val66Met polymorphism was differentiated with the NlaIII restriction enzyme and the product was electrophoresed in 2% agarose gels and stained with ethidium bromide and visualized on a UV trans-illuminator.

### Measurement of serum BDNF, glycosylated haemoglobin A1c, blood urea nitrogen, Cr: creatinine, UMA:urine micro- albumin

Blood samples were drawn into glass tubes without anticoagulants, which were immediately spun at 3000 rpm for 10 min at 4°C. Serum was isolated and stored at −20°C. The serum BDNF levels were measured by commercial ELISA kit (Adlitteram diagnostic laboratories, USA). After thawing, samples were centrifuged at 10,000 g for 10 min at 4°C for complete platelet removal. All samples and standards were measured in duplicate, and the coefficient of variation was less than 5%.

Plasma levels of blood urea nitrogen (BUN), Cr: creatinine (Cr) and urine micro- albumin (UMA) were measured using routine laboratory methods. Plasma glycosylated haemoglobin A1c (HbA1c) were measured using High-performance liquid chromatography (HA-8160).

### Statistical Analyses

Statistical analyses were performed using the SPSS Windows version 18.0. Genotype and allele frequency distributions were compared using the χ2 test. Hardy-Weinberg equilibrium was computed to the expected genotype distributions. Data was expressed as mean ± SD. The means for variables between the two groups were compared by a two-tailed unpaired Student’s t-test. Statistics among the three groups were analyzed by one-way ANOVA followed by post hoc individual comparisons with the SNK test and chi-square analysis for categorical variables. The associations of BDNF polymorphism and serum levels with the relevant clinical parameters and depression scale scores were analyzed by Pearson correlation and multiple step logistic regression. In both Pearson correlation and multiple step logistic regression, Met/Met + Met/Val were delegated by 1 and Val/Val was delegated by 2. All reported p values were two-tailed, and p values of < 0.05 were considered statistically significant.

## Results

### Participants and clinical characteristics in DDM group

In this study, we totally recruited 296 T2DM Han Chinese patients and 70 age- and gender-matched healthy subjects (mean age: 53.2 ± 5.5 vs. 55.2 ± 6.5 years, gender ratio: M/F 34/36 vs. 148/148) with the same ethnicity. Within the T2DM group, 64 patients (21.6%) had depression with 19 (12.8%) males and 45 (30.4%) females. In addition to a higher female/male ratio, the DDM group had higher HbA1c, BMI, educational levels and with more diabetic complications than the NDDM group (Table 1). There were no statistical differences in age, duration and marital status between the DDM group and the NDDM group (data not shown).

**Table 1:** Characteristics of NDDM and DDM group

**Variables**	**NDDM group**	**DDM group**	**p value**
	**(n = 232)**	**(n = 64)**	
Gender(M/F)	129/103	19/45	p < 0.001
Age (year)	53.1 ± 4.9	54.3 ± 5.7	p = 0.337
Education Level (%)			
≤Primary school	35(15.1%)	9(14.1%)	p = 1.000
Junior middle school	144(62.1%)	34(53.1%)	p = 0.199
Senior middle school	38(16.4%)	9(14.1%)	p = 0.847
Disease duration (year)	5.2 ± 1.3	6.2 ± 0.9	p = 0.234
The number of complications (n)	2.6 ± 0.8	5.1 ± 1.2	**p = 0.004**
BMI (kg/m^2^)	25.9 ± 3.8	28.3 ± 4.3	**p = 0.017**
HbA1c(%)	8.02 ± 1.88	8.91 ± 1.84	**p < 0.001**

Data expressed as mean ± S.D. or frequency [n (%)], BMI: body mass index; HbA1c: glycosylated haemoglobin A1c, p value < 0.05 for statistical significance.

### Association of BDNF Val66Met polymorphism with type 2 diabetes and depression

To assess whether BDNF Val66Met polymorphism contributed to depression in T2DM, 70 healthy subjects, 64 T2DM with depression and 232 T2DM without depression were genotyped. The genotype distributions of BDNF Val66Met followed Hardy-Weinberg equilibrium among the groups, were summarized in Table 2. The genotype distributions and allele frequencies of BDNF Val66Met were summarized in Table 3. Our study indicated that BDNF Val66Met polymorphism had no association with T2DM. However, within the T2DM group, there were statistical differences in genotype distributions and allele frequencies between the NDDM group vs. DDM group, p < 0.05. The DDM group carried higher frequencies of Met allele (53.9%, p < 0.05) compared to NDDM group (38.8%).

**Table 2:** Hardy-Weinberg equilibrium of BDNF Val66Met

**Group**	**n**	**A (T)**			**χ**^**2**^	**p**
		**Val/ Val**	**Val/Met**	**Met/Met**		
HC	70	26(25.8)	33(33.4)	11(16.6)	0.90	p > 0.05
NDDM	232	88(86.9)	108(110.7)	36(34.9)	0.05	p > 0.05
DDM	64	15(13.6)	29(31.8)	20(18.6)	0.23	p > 0.05

A is the actual number of genotypes; T is the expected number of genotypes. HC: Healthy control, NDDM: type 2 diabetes without depression group, DDM: type 2 diabetes with depression group.

**Table 3:** Genotype distributions of BDNF Val66Met

**Group**	**n**	**Genotype n (%)**			**Met allele frequency**
		**Val/ Val**	**Val/Met**	**Met/Met**	**Met(A)(%)**
HC	70	26(37.1)	33(47.1)	11(15.7)	39.3
NDDM	232	88(37.9)	108(46.6)	36(15.5)	38.8
DDM	64	15(23.4)	29(45.3)	20(31.3)	53.9

Data expressed as frequency [n (%)], p value < 0.05 for statistical significance, chi-square analysis used to identify differences in genotype frequencies between groups. HC: Healthy control, NDDM: type 2 diabetes without depression group, DDM: type 2 diabetes with depression group.

### Association of serum BDNF levels and BDNF genotypes

To explore whether serum BDNF levels was associated with BDNF Val66Met polymorphism, we divided the participants into 3 groups according to genotype distribution: Val/Val group, Val/Met group and Met/Met group. Our study revealed that there were significantly genotype-related differences in serum BDNF levels among 3 groups (F = 7.39, p < 0.05, Table 4). Interestingly, Val/Val carriers had the highest serum BDNF levels and Met/Met carriers had the lowest serum BBNF levels.

**Table 4:** Serum BDNF levels in three genotypes

**Genotype**	**n**	**Serum BDNF level(pg/ml)**	**F**	**p value**
Val/ Val group	129	83.83 ± 3.97	F = 7.39	p < 0.05
Val/Met group	170	79.04 ± 5.19*		
Met/Met group	67	76.59 ± 5.12*#		

Data expressed as mean ± S.D., p value < 0.05 criteria for statistical significance. *p < 0.05 vs. Val/Val group, # p < 0.05 vs. Val/Met group.

### Association of serum BDNF levels in patients with T2DM and depression

There were significant differences in serum BDNF levels in HC, NDDM and DDM groups (F = 24.94, p < 0.01, Table 5). NDDM subjects had significantly lower serum BDNF levels compared to HC (p < 0.05), but had significantly higher serum BDNF levels when compared to DDM subjects (p < 0.05).

**Table 5:** Serum BDNF levels in T2DM and depression subjects

**Group**	**n**	**Serum BDNF level(pg/ml)**	**F**	**p value**
HC	70	85.29 ± 3.27	F = 24.94	**p < 0.01**
NDDM	232	79.84 ± 5.15*		
DDM	64	76.67 ± 5.35*#		

Data expressed as mean ± S.D., p value < 0.05 criteria for statistical significance. * P < 0.05 vs. HC group, # P < 0.05 vs. NDDM group.

### Association of BDNF Val/Met polymorphism and serum levels with T2DM clinical characteristics and depression

Among type 2 diabetes subjects, multiple stepwise regression analysis revealed that when BDNF Val/Met genotypes against depression scores, gender, BMI, HbA1c, BUN, Cr and numbers of complications, were independently correlated with depression scores (OR = 1.952, **p < 0.05**) and HbA1c (OR = 1.786, **p < 0.05**), indicating that the Met allele contributed to both the risk of development depression and diabetes. Moreover, Pearson correlation analysis showed that when BDNF serum levels were tested for simple linear correlations against genotypes, depression scores, gender, BMI, HbA1c, BUN, Cr and numbers of complications, significant inverse correlations were found with genotypes (r = −0.346, **p < 0.01**), depression scores (r = −0.416, **p < 0.01**) and HbA1c (r = −0.168, **p < 0.05**), indicating that low BDNF concentrations may be a pathogenic factor associated with both depression and T2DM. Furthermore, after adjustment the presence or absence risk factors for depression including gender, HbA1c, BMI , BUN(μmol/l), Cr(μmol/l) and numbers of complications, BDNF Val/Met genotype distributions (OR = 2.105, **p < 0.05**) and decreased serum levels of BDNF (OR = 0.835, **p < 0.01**) were also independently associated with depression in T2DM (Table 6).

**Table 6:** Analysis of risk factors for depression in type 2 diabetes patients

	**B**	**S.E.**	**Wald**	**P**	**Exp(B)**	**95%C.I.forExp(B)**	
					**(OR)**	**Lower**	**Upper**
Female	1.035	0.294	12.412	0.000	2.815	1.583	5.008
Education level (n)	0.274	0.168	2.667	0.102	1.316	0.947	1.829
HbA1c(%)	1.792	0.219	4.251	0.041	1.885	1.709	2.987
BMI(kg/m2)	−0.062	0.038	2.463	0.117	0.942	0.874	1.015
SBP(mmHg)	0.006	0.011	0.263	0.608	1.006	0.984	1.028
DBP(mmHg)	0.001	0.019	1.006	0.938	1.002	0.964	1.040
UMA(μmol/l)	0.002	0.003	0.368	0.544	1.017	0.996	1.007
BUN(μmol/l)	0.281	0.093	9.204	0.002	1.324	1.015	1.558
Cr(μmol/l)	0.027	0.008	10.679	0.001	1.373	0.958	1.989
Number of complications(n)	2.513	0.534	2.486	0.024	1.094	0.907	1.142
Val → Met	3.114	1.272	10.434	0.035	2.105	1.282	4.991
BDNF(pg/ml)	−1.181	0.064	7.856	0.005	0.835	0.736	0.947

These variables were tested by multiple step logistic regression analysis. HbA1c: glycosylated haemoglobin A1c , BMI: body mass index, SBP: systolic blood pressure, DBP: diastolic blood pressure, BUN: blood urea nitrogen, Cr: creatinine, UMA:urine micro- albumin, BDNF: Brain-derived neurotrophic factor.

## Discussion

In the present study, we investigated the association of BDNF Val66Met polymorphism with depression in T2DM of Chinese Han subjects. Our results show that, for Chinese subjects, there is a positive association between BDNF Val66Met polymorphism and comorbid depression in T2DM patients and Met allele carriers are susceptible to suffer from depression. Our data also show that the serum BDNF levels are decreased in T2DM patients carrying either 66Met/Met homozygote or 66Val/Met heterozygote compared to those carrying 66Val homozygote. We also found a significant inverse association between BDNF levels and HbA1c.

The functional Val66Met polymorphism of BDNF has been studied for its possible associations with depression, but reports were contradictory. Hong et al. first reported that BDNF Val66Met polymorphism was associated with major depression disorders in a Caucasian population [13,15]. Subsequently, increasing clinical studies have confirmed that the Met allele was found more often among individuals with mood disorders [16-18]. Paradoxically, Tsai SJ et al. reported a higher incidence of depression in Val, not Met carriers [20]. In contrast, the meta-anlysis done by Verhagen et al. did not detect any significant genetic association between the BDNF Val66Met polymorphism and major depression disorders [38]. Differences between our study and previous study findings of an association between the BDNF Val66Met polymorphism and depression may be due to ethnic variances and alternative definitions for depression. In this study, we were among the first to identify that there is no association between BDNF Val66Met polymorphism and T2DM in Chinese subjects. Yet, we have demonstrated a significant association between the BDNF Val66Met polymorphism and depression within the T2DM group. We found that DDM group carried a higher frequency of Met allele (53.9%). After adjustment for age, gender, BMI and HbA1c, BDNF Val/Met genotypes were still independently correlated with depression scores (OR = 1.952, p < 0.05). Our study provides further evidence that the BDNF Val66Met polymorphisms may be a good therapeutic candidate gene for T2DM patients with depression in Chinese subjects.

The prevalence of depression is increasing at a significantly greater rate in patients with T2DM as compared with people without diabetes [39]. How could the BDNF Val66Met polymorphism influence depression in the T2DM subjects? Probable explanation is that the Val66Met polymorphism in the BDNF gene has been shown to impact secretion of BDNF [13]. A number of studies have suggested that the Met allele is associated with poorer episodic memory performance, a symptom often observed in subjects with MDD. Furthermore, it has been shown that the Met allele is associated with reduced hippocampal activity [13]. Studies in animal models have shown that the presence of the Met allele alters the sorting and secretion of proBDNF, such that less regulated secretion is likely to occur in carriers of at least one Met allele [40]. In this study, the serum BDNF levels are significantly lower in T2DM patients carrying either 66Met homozygote or 66Val/Met heterozygote compared with that carrying 66Val homozygote, indicating that this homozygosity for the 66Met allele confers an increased risk for depression in T2DM patients through mechanism involving reduced BDNF production and secretion. The study by Ribeiro et al. has also shown that Val66Met variants were associated with depression, presuming that the variants might affect the genesis and secretion of BDNF [13,15]. Moreover, Krabbe et al. discovered that the lower level of BDNF accompanied T2DM established a negative correlation between BDNF levels and fasting blood glucose. In other words, as fasting blood glucose progressively increased, the output of BDNF from the brain is progressively decreased. Collectively, the decreased BDNF may be a pathogenetic factor involved not only in depression, but also in T2DM [32]. In this study, we also found that BDNF serum levels were inversely correlated with HbA1c as a reflection of the average level of blood glucose over the previous 3 months. Therefore, we suggest that the decreased levels of BDNF can not be solely ascribed to Val66Met variants and the influence of elevated glucose level should also be considered.

Regarding other potential factors involved in T2DM with depression, we also found that HbA_1_c, BUN, Cr and the number of complications were independent risk factors for comorbid depression in T2DM patients, confirming that diabetes and depression interacted with each other [1]. First, depression may have resulted from the biologic stress of living with diabetes. This is because the biochemical changes associated with diabetes such as stress, inflammation, hyperglycemia and activations of the hypotha-lamic pituitary adrenal axis which induce arousal of the nervous system and may lead to the development of depression [39]. Moreover, depression may also results from the burden of living with T2DM such as poor diet, physical inactivity and taking insulin or from the psychological stress while coping with diabetes [41,42]. Furthermore, poor glycemic control is associated with elevated plasma concentrations cortisol and greater sensitivity to stress, possibly leading to concurrent diabetes and depression. Poor glycemic control may also adversely affects mood, and further aggravates depression. On the other hand, depression also contributes to the development of diabetes. Prospective studies have observed that many unhealthy lifestyles occur in patients with depression, such as binge eating and lack of exercise, which also increase diabetes risk [43]. Depression induces increased production of corticosteroids and catecholamine by affecting neuroendocrine system, especially the function of hypothalamus-pituitary-adrenal axis and central nerve, which, in turn, influence blood glucose indirectly [44]. Taken together, these studies provide some evidences to support the strong association between T2DM and depression.

There are two other issues noted in our study that might contribute to the development of depression in T2DM. First is the gender difference in the prevalence of depression occuring in 30.4% in women and 12.8% in men, in line with the findings from a previous study [45]. Interactions between the BDNF Val66Met polymorphism and environments may differ between men and women. Although the amount of life events men and women experience is rather similar, several studies have indicated that women subjectively experience more stress and have an increased stress response even they have the same BDNF Val66Met polymorphism [46]. The gender-related influence of the BDNF Val66Met polymorphism in depression may be due to sexual dimorphism in brain structures involved in the neurobiology of depression, particularly, the hippocampus. Male and female hippocampi have been documented to differ significantly in their anatomical structure, their neurochemical make-up and their reactivity to stressful situations [47]. The second is that education levels are related to depression. Consistent with our study, Abolfotouh el al suggested that lower education levels are associated with an increased incidence of depression [48].Whereas other studies provided evidence that the higher educated people are more likely to develop depression [49].

In conclusion, our current study demonstrated that the presence of the functional BDNF Val66Met polymorphism was associated with depression in T2DM by decreasing the serum BDNF levels, suggesting that increased BDNF levels may be a therapeutic potential in depression patients with T2DM. Limitations of the present study are that our sample was not large for a genetic polymorphism, and we did not perform any interventions on our patients. A large primary study will be necessary to confirm our study and validate the therapeutic response in the depression with T2DM patients who carry Met allele.

## Competing interests

The authors have declared that they have no conflicts of interests.

## Authors' contributions

Chen LM and Zhou JX conceived the study, analyzed data and wrote the manuscript. Bai XJ and Li HC acquired and analyzed data, and Chang BC, Li CJ and Sun P wrote the manuscript. All authors read and approved the final manuscript.
